# Standardized Workflow for the Generation of Patient-Derived Glioblastoma Spheroids

**DOI:** 10.3390/mps9020061

**Published:** 2026-04-03

**Authors:** Giuseppa D’Amico, Alessandra Maria Vitale, Martina Di Marco, Alessandro Lo Giudice, Francesca Chiara Cecala, Francesco Cappello, Celeste Caruso Bavisotto

**Affiliations:** 1 Institute of Human Anatomy and Histology, Department of Biomedicine, Neurosciences and Advanced Diagnostics, University of Palermo, 90133 Palermo, Italy; martina.dimarco@unipa.it (M.D.M.); alessandro.logiudice03@unipa.it (A.L.G.); francescachiara.cecala@community.unipa.it (F.C.C.); francesco.cappello@unipa.it (F.C.); 2 Euro-Mediterranean Institute of Science and Technology (IEMEST), 90139 Palermo, Italy

**Keywords:** glioblastoma, primary cell culture, 2D model, 3D model, spheroid, immunofluorescence

## Abstract

Glioblastoma (GBM) is one of the most aggressive and therapy-resistant primary brain tumors, mainly due to its pronounced intratumoral heterogeneity and highly invasive phenotype. Patient-derived three-dimensional (3D) culture models, including tumor spheroids, represent valuable tools to preserve the cellular complexity, phenotypic plasticity, and microenvironmental features of GBM ex vivo. However, standardized and reproducible protocols for the generation and maintenance of GBM spheroids from surgical specimens are still limited. Here, we describe a detailed and robust protocol for the isolation, 3D cultures, and expansion of primary GBM cells obtained from patient biopsies, leading to the formation of stable and morphologically consistent spheroids. The protocol provides step-by-step instructions for tissue dissociation, cell seeding under low-adhesion conditions, optimization of culture density, and long-term spheroid maintenance. In addition, we include guidelines for the morpho-phenotypical characterization of the resulting 3D structures. This methodological workflow offers a reproducible platform for modeling GBM in vitro, enabling the study of tumor biology and supporting translational applications such as drug screening, biomarker validation, and patient-specific therapeutic testing in a 3D context.

## 1. Introduction

Glioblastoma (GBM) is the most aggressive and lethal primary tumor of the central nervous system, characterized by rapid progression, extensive cellular heterogeneity, diffuse infiltration into the surrounding brain parenchyma, and marked resistance to conventional therapies [1]. Despite decades of research and multimodal treatment strategies, including surgery, radiotherapy, and chemotherapy, the median survival of GBM patients remains approximately 15 months, highlighting the urgent need for more predictive experimental models and innovative therapeutic approaches [2].

One of the major biological features underlying therapeutic failure in GBM is its profound intratumoral heterogeneity, which encompasses genetic, epigenetic, phenotypic, and functional diversity among tumor cell subpopulations [3,4]. This heterogeneity is further shaped by complex interactions between tumor cells and the surrounding microenvironment, including hypoxia, extracellular matrix components, and non-neoplastic stromal and immune cells [5,6,7,8,9,10]. Traditional two-dimensional (2D) monolayer cultures fail to adequately recapitulate these features, as they impose artificial growth conditions that profoundly alter cell morphology, polarity, metabolism, and response to therapies [11,12,13].

In recent years, three-dimensional (3D) in vitro models, including tumor spheroids and organoid-like cultures, have emerged as more physiologically relevant systems to model tumor architecture, cellular interactions, and diffusion gradients of oxygen and nutrients [14,15,16,17,18]. In the context of GBM, patient-derived spheroids have been shown to better preserve the histopathological, molecular, and functional characteristics of the original tumors compared to conventional cell lines [19,20,21]. These models have proven particularly valuable for studying tumor invasion, therapy resistance, and for testing experimental drugs in a patient-specific manner [22,23,24,25]. Nevertheless, the field still lacks broadly accepted and standardized protocols for the reliable generation, maintenance, and characterization of GBM spheroids from surgical specimens, which limits inter-laboratory reproducibility and translational scalability [26,27].

Several methodological variables (including tissue dissociation strategies, culture conditions, seeding density, and long-term maintenance procedures) can significantly influence spheroid formation efficiency, structural stability, and cellular composition [26,28]. Moreover, while many studies focus on spheroid generation, fewer provide detailed and reproducible workflows for their systematic morphological and phenotypic characterization, which is essential to validate their biological relevance and experimental robustness [29,30]. This methodological heterogeneity represents a critical bottleneck for the widespread adoption of patient-derived 3D GBM models in both basic and translational research.

In this work, we present a detailed, standardized, and reproducible protocol for the isolation of primary GBM cells from surgical biopsies, their assembly into stable 3D spheroids under low-adhesion conditions, and their subsequent maintenance and expansion over time. In addition, we provide a step-by-step workflow for the morpho-phenotypical characterization of the resulting 3D structures. This protocol is designed to offer a robust and accessible platform for modeling GBM in vitro, supporting applications ranging from basic tumor biology studies to drug screening and patient-specific therapeutic testing in a 3D context.

## 2. Experimental Design

The following protocol provides a detailed description of the required materials and step-by-step procedures for performing the isolation of biopsy-derived primary cells, the subsequent formation of a spheroid-based 3D culture model that mimics the in vivo tumor architecture, and the single or double immunofluorescence staining on whole spheroids, enabling the identification of specific molecular markers and/or the visualization of cellular niche formation within the 3D micro-architecture.

### 2.1. Materials

- 1X Dulbecco’s Phosphate-Buffered Saline (DPBS) *w*/*o* Ca^2+^ and Mg^2+^ (Dutscher, Bernolsheim, France, Cat. no.: L0615);
- Phosphate-Buffered Saline (PBS) (Gibco—Themo Fischer Scientific, Waltham, MA, USA, Cat. no.: 18912-014);
- 1X Dulbecco’s Modified Eagle Medium (DMEM) (Corning, Corning, NY, USA, Cat. no.: 10-013-CV);
- Euromed-N Medium (Euroclone, Pero (MI), Italy, Cat. no.: ECM0883L);
- Nutrient Mixture F-12 Ham (Gibco, Cat. no.:21765-029);
- Heat-inactivated Fetal Bovine Serum (FBS) (Sigma-Aldrich, St. Louis, MO, USA, Cat. no.: F7524);
- L-Glutamine 2 mM (Corning, Cat. no.: 25-005-CI);
- Penicillin 100 U/mL (Lonza Bioscience, Walkersville, MD, USA, Cat. no.: DE17-602E);
- Streptomycin 100 μg/mL (Lonza Bioscience, Cat. no.: DE17-602E);
- Antibiotic-antimycotic (100X) (Gibco, Cat. no.: 15240062);
- Trypsin/EDTA Solution 100X (Gibco, Cat. no.: R001100);
- Type II collagenase 250 U/mL (Gibco, Cat. no.: 17101015);
- MEM Non-Essential Amino Acids Solution (100X) (Gibco, Cat. no.: 11140050);
- Agarose powder (Sigma-Aldrich, Cat. no.: A5093);
- 4% paraformaldehyde (PFA) (*w*/*v*) in 1X PBS (Thermo Fisher Chemicals—Themo Fischer Scientific, Cat. no.: 416785000);
- 3% BSA (*w*/*v*) in 1X PBS, i.e., blocking solution (Dutscher, Cat. no.: P6154);
- Sodium Citrate Buffer, i.e., unmasking solution (10 mM trisodium citrate, 0.05% Tween 20, pH 6) (Sigma Aldrich, Cat. no.: S4641);
- Primary antibodies: mouse anti-PCNA, used at 1:50 for IF (Santa Cruz Biotechnology, Santa Cruz, California, USA, Cat# sc-25280, RRID:AB_628109); rabbit anti-AIF, used at 1:50 for IF (Santa Cruz Biotechnology Cat# sc-5586, RRID:AB_2224668);
- Fluorochrome-conjugated secondary antibodies diluted 1:100 in 1X PBS: anti-rabbit IgG Atto 647N antibody produced in goat, 40839, Sigma-Aldrich, St. Louis, MO, USA; anti-mouse IgG-Atto 488 antibody produced in goat, 62197, Sigma-Aldrich, St. Louis, MO, USA; anti-rabbit IgG (whole molecule)-FITC antibody produced in goat, F0382, Sigma-Aldrich, St. Louis, MO, USA; anti-mouse IgG (whole molecule)-TRITC antibody produced in goat, T5393, Sigma-Aldrich, St. Louis, MO, USA;
- DAPI (4′,6-diamidino-2-phenylindole) (Sigma-Aldrich, Cat. no.: D9542);
- Quick-hardening mounting media (e.g., Eukitt^®^) (Sigma-Aldrich, Cat. no.: 03989).

### 2.2. Equipment

- Biological safety cabinet;
- Chemical hood;
- 60 mm Nunc™ EasYDish™ plates (Thermo Scientific—Thermo Fischer Scientific, TM: 150462);
- 100 mm Nunc™ EasYDish™ plates (Thermo Scientific TM: 150466);
- 25 cm^2^ Nunc™ EasYFlask™ flasks (Thermo Scientific Cat. no.: 156367);
- Incubator humidified at 5% CO_2_ and 37 °C;
- Scalpel;
- BD Falcon™ 70 μm Cell Strainer (Product Number: 352350);
- 15 mL Polypropylene conical tubes (Falcon—Corning; Cat. no.: 352096);
- 50 mL Polypropylene conical tubes (Falcon; Cat. no.: 352070);
- 100–1000 μL and 20–200 μL pipettes and corresponding tips;
- Polyethylene Pasteur pipettes;
- 1.5 mL Microcentrifuge tubes;
- Benchtop centrifuge;
- Orbital shaker;
- Burker chamber;
- Microwave oven;
- Thermoblock;
- Tissue culture plate, 96-well, flat bottom;
- Hydrophobic pap pen;
- Glass microscope slides and coverslips;
- Confocal microscope (Leica Confocal Microscope TCS SP8; Leica Microsystems, Heidelberg, Germany);
- Inverted phase-contrast microscope.

## 3. Procedure

**Figure 1:**
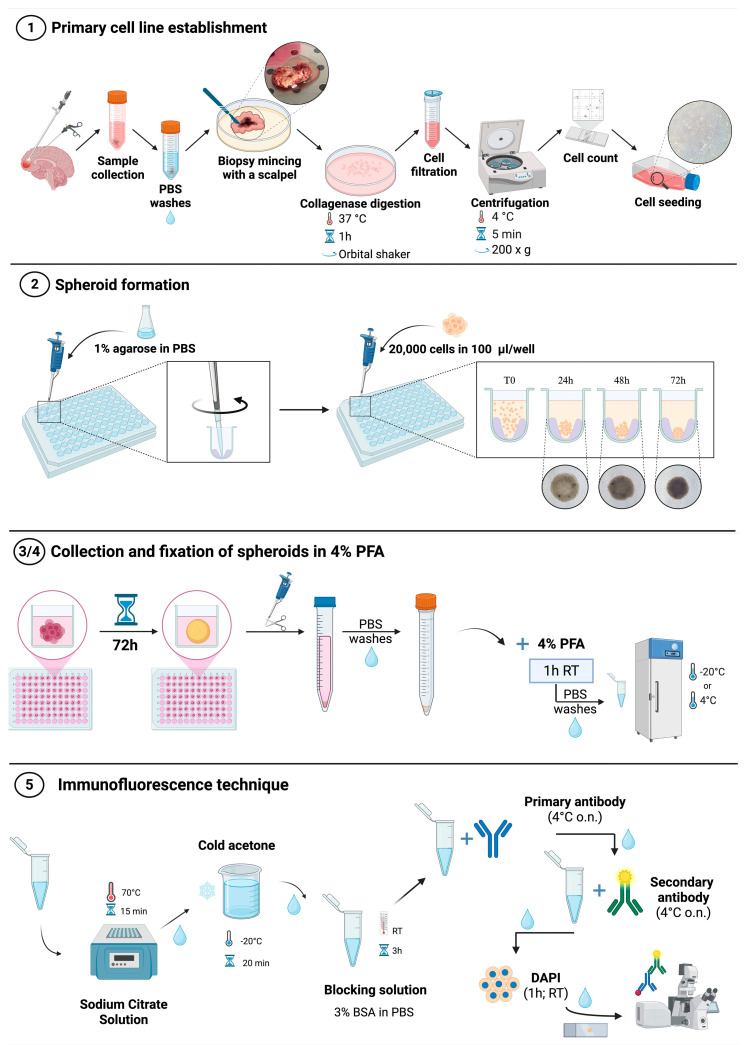
Illustrated steps regarding the cutting of the biopsy and the isolation of primary cells (**1**), the subsequent formation of spheroids with the “liquid-overlay” method (**2**), their collection and fixation in 4% PFA (**3/4**) and the immunofluorescence technique applied to whole spheroids (**5**). Created in Biorender. Bucchieri, F. (2026). https://BioRender.com/263pddd (accessed on 27 January 2026).

The procedures concerning the isolation of primary cells from biopsy specimens, the formation of 3D spheroid-based culture models, their collection and fixation, and subsequent analysis by immunofluorescence are summarized in Figure 1.

### 3.1. Primary Cell Line Establishment (~2 h)

1. Collect the bioptic sample inside an appropriate medium (1X DMEM supplemented with 100 U/mL penicillin, 100 μg/mL streptomycin and 1X antibiotic-antimycotic 100X);
2. In a biological safety cabinet, wash the biopsy, using 1X DPBS *w*/*o* Ca^2+^ and Mg^2+^, to eliminate any traces of blood;
3. Cut the biopsy with a scalpel into small pieces (~1–5 mm) on a 100 mm plate;
4. Incubate the obtained small pieces in an orbital shaker at 37 °C for 1 h on a 60 mm plate containing 5 mL of DMEM supplemented with 2 mM L-Glutamine, 100 U/mL penicillin, 100 μg/mL streptomycin, and 250 U/mL collagenase type II;
5. Filter the cell suspension with a 70 μm cell strainer into a 50 mL conical tube to collect cells;
6. Centrifuge the tube containing the cell suspension at 200× *g* for 5′ at 4 °C;
7. Resuspend the cell pellet with the appropriate medium: 50% Euromed-N Medium, 50% 1X DMEM:F-12, 10% FBS, 1% L-Glutamine, 1% penicillin/streptomycin, 1% Non-Essential Amino Acids;
8. Seed the cells into T25 flasks (~5 × 10^5^ cells/flask) and place in a humidified atmosphere of 5% CO_2_ at 37 °C;
9. Monitor GBM primary cells using an optical phase-contrast microscope (Nikon ECLIPSE Ti2, Amstelveen, The Netherlands, EU);
10. Passage GBM primary cells of use for downstream experiments when they reach an 80% of confluency.

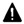

**CRITICAL STEP:** The biopsy cut should be performed as rapidly as possible, while continuously keeping the biopsy moist, in order to prevent necrotic cell death. Be sure to remove all blood from the biopsy to improve isolation yield. The efficiency of primary GBM cell isolation depends primarily on the cellularity and viability of the starting biopsy. In our experience, highly necrotic regions—often identifiable by a dark-red appearance—show reduced isolation yield and lower proliferative potential. For this reason, during the mechanical dissection step, visibly necrotic areas are carefully removed prior to enzymatic digestion to enrich for viable tumor cells. This pre-selection step improves reproducibility and consistency in primary culture establishment and subsequent spheroid formation.
  **NOTE**: The serum-containing culture conditions described in this protocol (50% Euromed-N Medium, 50% DMEM:F-12 supplemented with 10% FBS) are intended to support the expansion of differentiated bulk GBM cells and do not selectively enrich for glioblastoma stem-like cells (GSCs). The isolation and propagation of GSCs typically require serum-free defined media supplemented with specific growth factors, which promote spontaneous *neurosphere* formation.

### 3.2. 3D Cell Culture (Spheroid) Establishment (at Least 3 Days)

1. Prepare a 1% agarose solution by dissolving the agarose powder in 1X sterile PBS and heating in a microwave oven until totally dissolved (usually when the solution is boiling at about 100 °C). For instance, prepare a total of 10 mL of 1% agarose solution for each 96-well plate.
2. Using a 200 µL pipette, dispense 50 µL of agarose solution into each well of a 96-well plate.
3. Immediately after dispensing, gently perform circular movements to allow the formation of a low-attachment surface as illustrated in Figure 1.
4. Place the 96-well plate into an incubator humidified at 5% CO_2_ and 37 °C for at least 20 min.
5. Meanwhile, enzymatically detach the cells using 1X Trypsin/EDTA Solution in 1X DPBS *w*/*o* Ca^2+^ and Mg^2+^ for 5 min at 37 °C. Inactivate the trypsin solution by adding an equal amount of complete medium, collect and centrifuge for 5 min at 200× *g*. Resuspend the cell pellet in an appropriate amount of complete medium and count the GBM primary cells.
6. Seed 2 × 10^4^ cells into previous agarose-coated wells in the appropriate culture medium (100 μL/well).
7. Monitor the growth and formation of spheroids using an optical phase-contrast microscope.

**OPTIONAL STEP:** Upon cell seeding, a short initial centrifugation of the 96-well plate may facilitate cell aggregation. During the spheroid maintenance, add fresh culture medium if necessary.

### 3.3. Spheroid Collection and Washing (~10′–30′, Depending on the Number of Spheroids)

1. After at least 72 h of culturing, collect the 3D spheroids in a 15 mL conical tube using a 200 μL pipette in a biological safety cabinet;
2. Allow the 3D spheroids to settle spontaneously at the bottom of the 15 mL conical tube;
3. Carefully remove the supernatant with an appropriate pipette (100–1000 μL or 20–200 μL);
4. Add 1 mL of 1X DPBS *w*/*o* Ca^2+^ and Mg^2+^ and wash the spheroids by gently pipetting up and down;
5. Repeat the previous step until the supernatant appears clear.

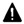

**CRITICAL STEP:** During collection, the 3D spheroids may be damaged by mechanical insults. To avoid this issue, cut off the end of the 200 μL tip, gently pipette, ensuring each spheroid is collected from the well, and release carefully at the bottom of the 15 mL tube, avoiding bubble formation.
  **OPTIONAL STEP:** If the spheroids do not settle spontaneously at the bottom of the tube after collection and each wash step, you can centrifuge them at 115× *g* for 5 min and carefully remove the supernatant, without disturbing the pellet.

### 3.4. Spheroid Fixation in 4% PFA (~1.5 h)

1. After the last wash, ensure that the 3D spheroids have pelleted at the bottom of the tube and carefully remove the supernatant;
2. Working under a chemical hood, resuspend the pellet in 1 mL of 4% PFA and incubate for 1 h at room temperature (RT);
3. After fixation, ensure that the spheroids have pelleted at the bottom of the tube and carefully remove and discard the 4% PFA completely;
4. Wash the pellet twice in 1 mL of sterile 1X PBS;
5. After the last wash, transfer the spheroids to a new 1.5 mL conical tube in 1 mL of sterile 1X PBS;
6. Proceed with the immunofluorescent staining.

  **OPTIONAL STEP:** If the spheroids do not settle spontaneously at the bottom of the tube after fixation and each wash step, you can centrifuge them at 115× *g* for 5 min and carefully remove the supernatant, without disturbing the pellet.

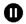

**PAUSE STEP:** Fixed samples can be stored at 4 °C for up to four months, or directly at −20 °C for a longer period.

### 3.5. Single or Double Immunofluorescence Analysis (3–5 Days)

**Figure 2:**
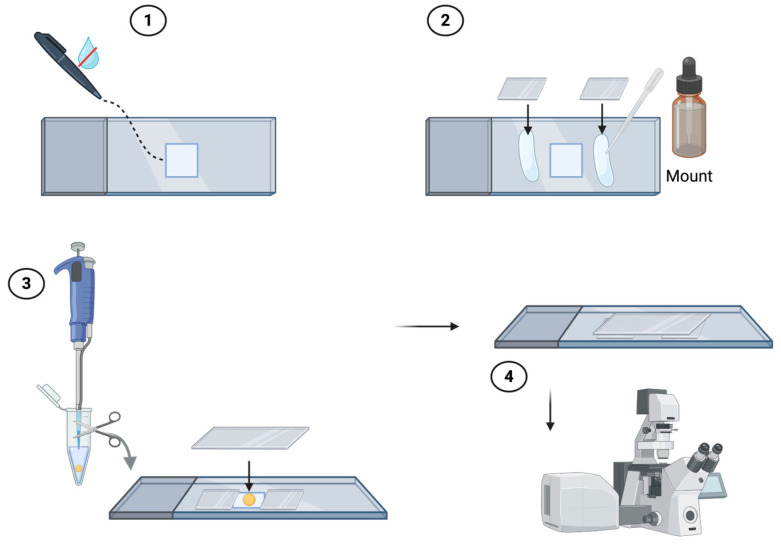
Steps for creating the chamber to observe the spheroids under a confocal microscope: (**1**) Create a square in the center of the slide using the hydrophobic pap pen; (**2**) Use an aqueous mount to position the coverslips at the edge of the square; (**3**) Spot the spheroids resuspended in PBS in the square and close the chamber with a coverslip placed on top; (**4**) Slide ready for analysis under a confocal microscope. Created in Biorender. Bucchieri, F. (2026). https://BioRender.com/263pddd (accessed on 27 January 2026).

1. Ensure that the spheroids are at the bottom of the tube, and then carefully remove the supernatant.
2. Gently add 0.5–1 mL of the unmasking solution, and incubate for 15 min at 70 °C. You can place the eppendorf tube containing the spheroids resuspended in the unmasking solution in a thermoblock at 70 °C.
3. Ensure that the spheroids are at the bottom of the tube, and then carefully remove the supernatant and rinse 2–5 times with 0.5–1 mL of 1X PBS.
4. Ensure that the spheroids are at the bottom of the tube, and then carefully remove the supernatant and gently add 0.5–1 mL of cold acetone for 20 min at −20 °C.
5. Ensure that the spheroids are at the bottom of the tube, and then carefully remove the supernatant and rinse 2–5 times with 0.5–1 mL of 1X PBS.
6. Ensure that the spheroids are at the bottom of the tube, and then carefully remove the supernatant, add 0.5 mL of blocking solution and incubate for 3 h at RT.
7. After the blocking, transfer the spheroids to different eppendorf tubes (always using a cut pipette tip) based on the number of primary antibodies to be incubated and considering one eppendorf tube for the negative control. Next, make sure that the spheroids are at the bottom of the tube, and then carefully remove the supernatant.
8. Add the primary antibody diluted in 1X PBS according to the manufacturer’s instructions and incubate overnight (o.n.) at 4 °C. In the negative control, incubate o.n. with 1X PBS only.
9. The next day, ensure that the spheroids are at the bottom of the tube, and then carefully remove the supernatant and rinse 1–5 times with 0.5–1 mL of 1X PBS.
10. Ensure that the spheroids are at the bottom of the tube, and then carefully remove the supernatant and incubate o.n. at 4 °C with an appropriate secondary fluorochrome-conjugated antibody diluted in 1X PBS according to the manufacturer’s instructions.
  Remember to add the secondary antibody to both the sample(s) and the negative control.
  If you perform a double immunofluorescence, carefully remove the supernatant and rinse 2–5 times with 0.5–1 mL of 1X PBS before repeating the previous three steps with a second primary antibody and a corresponding appropriate secondary fluorochrome-conjugated antibody.
11. On the last day, ensure that the spheroids are at the bottom of the tube, and then carefully remove the supernatant and rinse 2–5 times with 0.5–1 mL of sterile 1X PBS.
12. Add DAPI diluted 1:1000 in 1X PBS for 1 h at RT to counterstain the nuclei.
13. Ensure that the spheroids are at the bottom of the tube, and then carefully remove the supernatant and rinse 2–5 times with 0.5–1 mL of 1X PBS.
14. Prepare a chamber consisting of a slide and two (or more) coverslips to ensure that it is the same thickness as the spheroids to be analyzed. Construct the chamber using a hydrophobic pap pen and aqueous mount (e.g., Eukitt) as illustrated in Figure 2. Resuspend the spheroids in 1X PBS and spot them into the constructed chamber. Finally, close the chamber with a coverslip on top. The samples are now ready for observation under a confocal microscope.

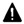

**CRITICAL STEP:** During the different steps, the spheroids may be damaged or lost. To minimize this risk, remove and add all volumes gently, avoiding bubble formation. Both the volumes and the number of washing steps can be adjusted as required.
  **OPTIONAL STEP:** If the spheroids do not settle spontaneously at the bottom of the tube at each step, you can centrifuge them at 115× *g* for 5 min and carefully remove the supernatant, without disturbing the pellet.

## 4. Expected Results

Using this standardized protocol, primary GBM cells derived from surgical specimens are expected to reproducibly generate compact and well-defined 3D spheroids under low-adhesion culture conditions. Spheroid formation should occur within a few days after seeding (3–5 days), with the progressive assembly of cohesive and morphologically regular structures. Under optimized conditions, spheroids are expected to maintain structural integrity and stable morphology over prolonged culture periods, supporting their use in medium- and long-term experiments.

The success rate of the entire protocol depends on the characteristics of the biopsy received, because if there are too many areas of necrosis, primary cells cannot be isolated. In general, we have observed a 70% yield using our procedure. Once the primary culture has stabilized, the success rate of spheroid formation is approximately 100%.

A key outcome of this workflow is the high reproducibility of spheroid generation across independent experiments and different patient-derived samples. When using identical seeding densities and culture conditions, spheroids are expected to display limited size variability and consistent growth kinetics, demonstrating the robustness and standardization of the procedure. Moreover, controlled modulation of the initial cell number should allow the generation of size-controlled spheroids, enabling experimental standardization according to specific downstream applications (e.g., imaging, drug testing, or molecular analyses).

**Figure 3:**
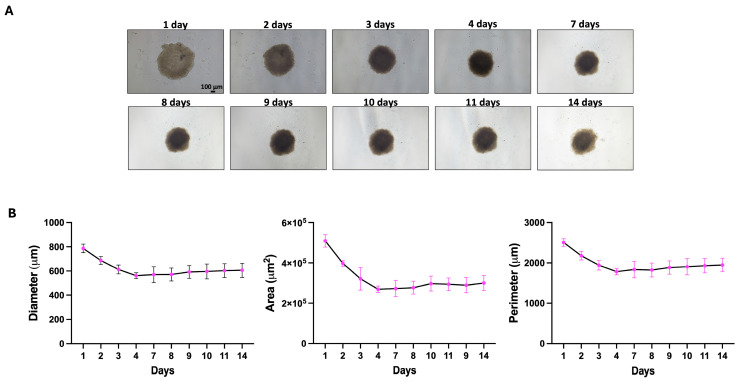
Long-term morphological evolution and quantitative characterization of patient-derived GBM spheroids generated from 20,000 cells. Representative bright-field images show the progressive morphological changes of spheroids cultured under low-adhesion conditions over a period of up to 14 days (scale bar: 100 µm) (**A**). Spheroids remain compact and structurally stable throughout the observation period. Quantitative analysis of spheroid diameter, area, and perimeter reveals an initial decrease in size during the early culture phase, followed by a progressive increase at later time points (**B**). This dynamic behavior reflects an initial phase of cellular compaction driven by the establishment of cell–cell interactions, followed by renewed growth and structural reorganization. At later stages, spheroids display a darker central core, consistent with the development of hypoxic regions, and an irregular, actively proliferating outer layer characterized by the presence of cytoplasmic protrusions suggestive of invasive behavior. Data are reported as mean ± SD.

Long-term culture of spheroids generated from 20,000 cells showed a reproducible and dynamic morphological evolution over a 14-day observation period. Quantitative analysis of diameter, area, and perimeter revealed an initial decrease in spheroid size during the early culture phase, followed by a progressive increase at later time points. This initial contraction likely reflects the progressive establishment of cell–cell interactions and tissue compaction, as also suggested by the transient appearance of a lighter central core surrounded by a denser outer layer. At later stages, once tight cellular cohesion is achieved, spheroids resume active growth and progressively develop structural gradients typical of 3D tumor models. In this phase, the central region becomes darker, consistent with the establishment of hypoxic conditions, while the outer layer appears irregular and composed of actively proliferating cells exhibiting cytoplasmic protrusions indicative of invasive behavior into the surrounding microenvironment (Figure 3).

We observed that spheroids remained viable and structurally stable for up to 14 days. Structural stability was defined as the maintenance of spheroid compactness, border integrity, and three-dimensional cohesion, without signs of fragmentation or structural collapse throughout the 14-day culture period.

From a structural and biological perspective, larger spheroids are expected to progressively develop a spatially organized architecture, typically characterized by a more proliferative outer region and a less oxygenated inner core, potentially including hypoxic or necrotic areas. This organization recapitulates key features of in vivo tumor tissue and represents an important indicator of the physiological relevance of the model. Cell viability is expected to be preserved for extended periods if spheroid size and culture conditions are properly controlled.

To further support this structural organization, representative paraffin-embedded spheroid sections were stained with hematoxylin and eosin (H&E) and are provided as Supplementary Material.

**Figure 4:**
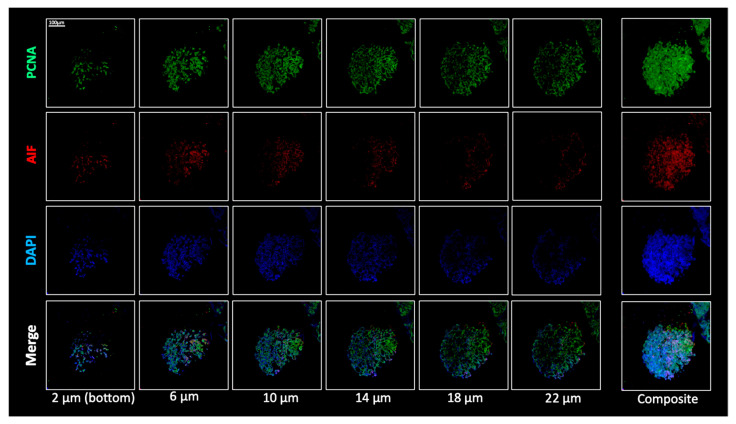
Morpho-phenotypical characterization of patient-derived GBM spheroids by confocal microscopy. Representative confocal images showing selected optical sections (z-stacks) acquired at different depths within the spheroid (2, 6, 10, 14, 18, and 22 µm from the bottom) and the corresponding composite reconstruction of the entire structure. Spheroids were stained for PCNA (Proliferating Cell Nuclear Antigen) (green), AIF (Apoptosis-Inducing Factor) (red), and nuclei (DAPI, blue). The images reveal a heterogeneous and spatially organized 3D architecture, with a non-uniform distribution of the analyzed markers across the spheroid volume, reflecting the intrinsic cellular heterogeneity of the model. PCNA-positive cells are predominantly localized in the outer layers of the spheroid, consistent with actively proliferating tumor cells, whereas AIF staining is mainly observed in the inner regions, indicating cells undergoing apoptosis within the central core. The composite view highlights the overall structural organization of the spheroid and confirms the suitability of the protocol for reproducible morpho-phenotypical characterization of patient-derived 3D tumor models. Scale bar: 100 µm.

Morpho-phenotypical analysis of patient-derived GBM spheroids by confocal microscopy is expected to reveal a complex and spatially organized three-dimensional architecture, characterized by a non-uniform distribution of glioblastoma-associated, proliferation- and phenotype-related markers across the spheroid volume. This heterogeneous spatial pattern reflects the intrinsic cellular diversity of the model and supports its biological consistency with respect to the original tumor tissue. Importantly, this staining and imaging workflow is expected to yield highly reproducible morphological and phenotypic readouts across independent experiments, further validating the robustness and standardization of the protocol for morpho-phenotypical characterization of 3D tumor models (Figure 4).

Overall, the spheroids generated using this protocol are expected to be robust, reproducible, and experimentally reliable 3D tumor models, suitable for a wide range of downstream applications, including drug screening, imaging-based assays, and molecular and functional studies. The emphasis on standardized procedures and controlled experimental parameters ensures that this workflow can serve as a reliable and transferable platform for patient-derived GBM modeling in different laboratories and experimental settings.

Unsatisfactory results may occur if cell self-aggregation is inefficient, with the formation of irregular or fragmented structures and excessive dispersion of individual cells. In light of this, in some cases, a brief initial centrifugation of the 96-well plate following cell seeding may facilitate cell aggregation. Both the speed and the duration of centrifugation may vary, depending on the shape and size of the primary cells. Therefore, these parameters must be carefully optimized to avoid cell stress, promote the formation of spheroids with comparable size and morphology, and ensure the reproducibility of the experiments.

## Data Availability

The original contributions presented in this study are included in the article/Supplementary Material. Further inquiries can be directed to the corresponding authors.

## Supplementary Materials

The following supporting information can be downloaded at https://www.mdpi.com/article/10.3390/mps9020061/s1, Videos and pictures of the procedures for primary cell line establishment (S1 and S2), 3D cell culture (spheroid) establishment (S3 and S4) and collection (S5), and H&E staining of paraffin-embedded spheroid sections (S6) can be found in the provided PowerPoint file. (S6) Representative sections of two spheroids showing preserved structural integrity and spatial heterogeneity. A panel: 20× magnification illustrating the overall spheroid architecture with a central region characterized by reduced cellular density and structural discontinuities. B panel: 40× magnification highlighting the distinction between the inner core and the peripheral layer. Arrows show migrating cells. C panel: higher magnification of the outer region showing elongated tumor cells, less condensed nuclei, and identifiable mitotic figures (arrow heads). These features are consistent with the presence of a hypoxic-like central core and a proliferative outer layer typical of three-dimensional tumor spheroids. Scale bar = 100 µm.

## Abbreviations

The following abbreviations are used in this manuscript:

2D	Two-dimensional
3D	Three-dimensional
BSA	Bovine Serum Albumin
DAPI	4′,6′-diamidino-2-phenylindole
DMEM	Dulbecco’s Modified Eagle Medium
DPBS	Dulbecco’s Phosphate-Buffered Saline
FBS	Fetal Bovine Serum
GBM	Glioblastoma
PBS	Phosphate-Buffered Saline
PFA	Paraformaldehyde
RT	Room temperature
