# Supplementary figures and images for: Standardized Workflow for the Generation of Patient-Derived Glioblastoma Spheroids
Source: Methods Protoc. 2026 Apr 3;9(2):61. doi: 10.3390/mps9020061 (PMC13118598; doi:10.3390/mps9020061)

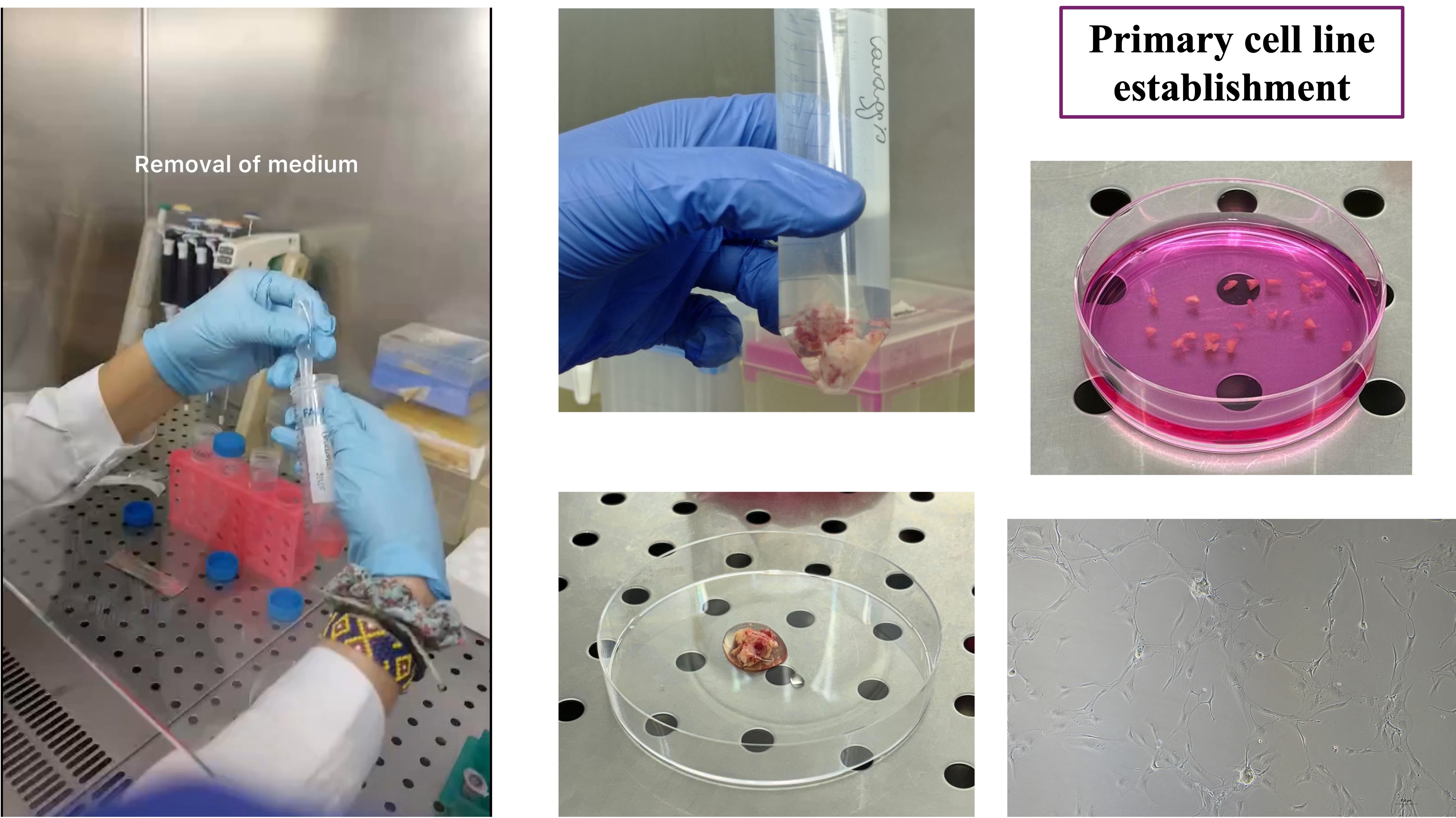

Supplement: Supplementary file 1 [file mps-09-00061-s001.zip › S2.jpg]

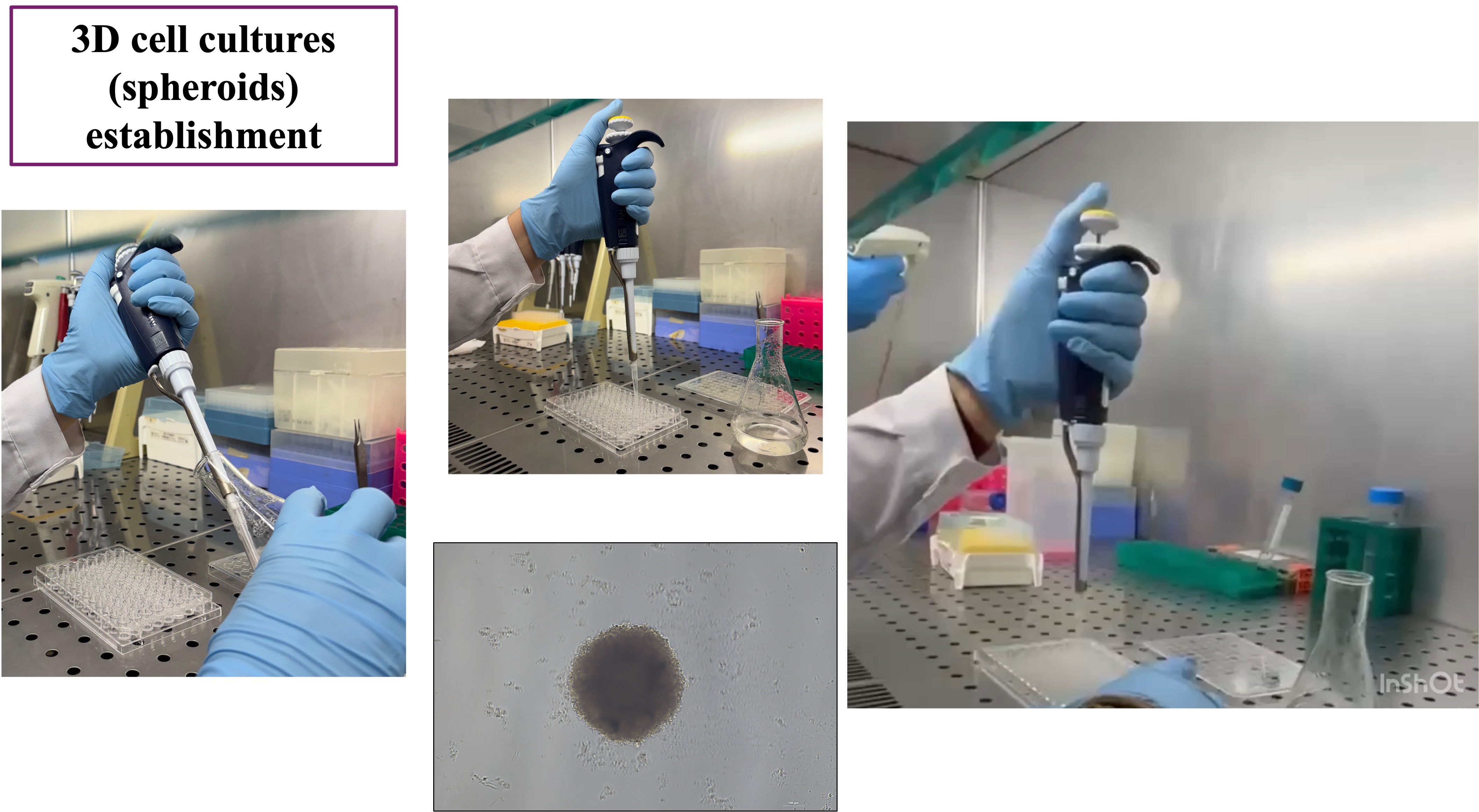

Supplement: Supplementary file 1 [file mps-09-00061-s001.zip › S3.jpg]

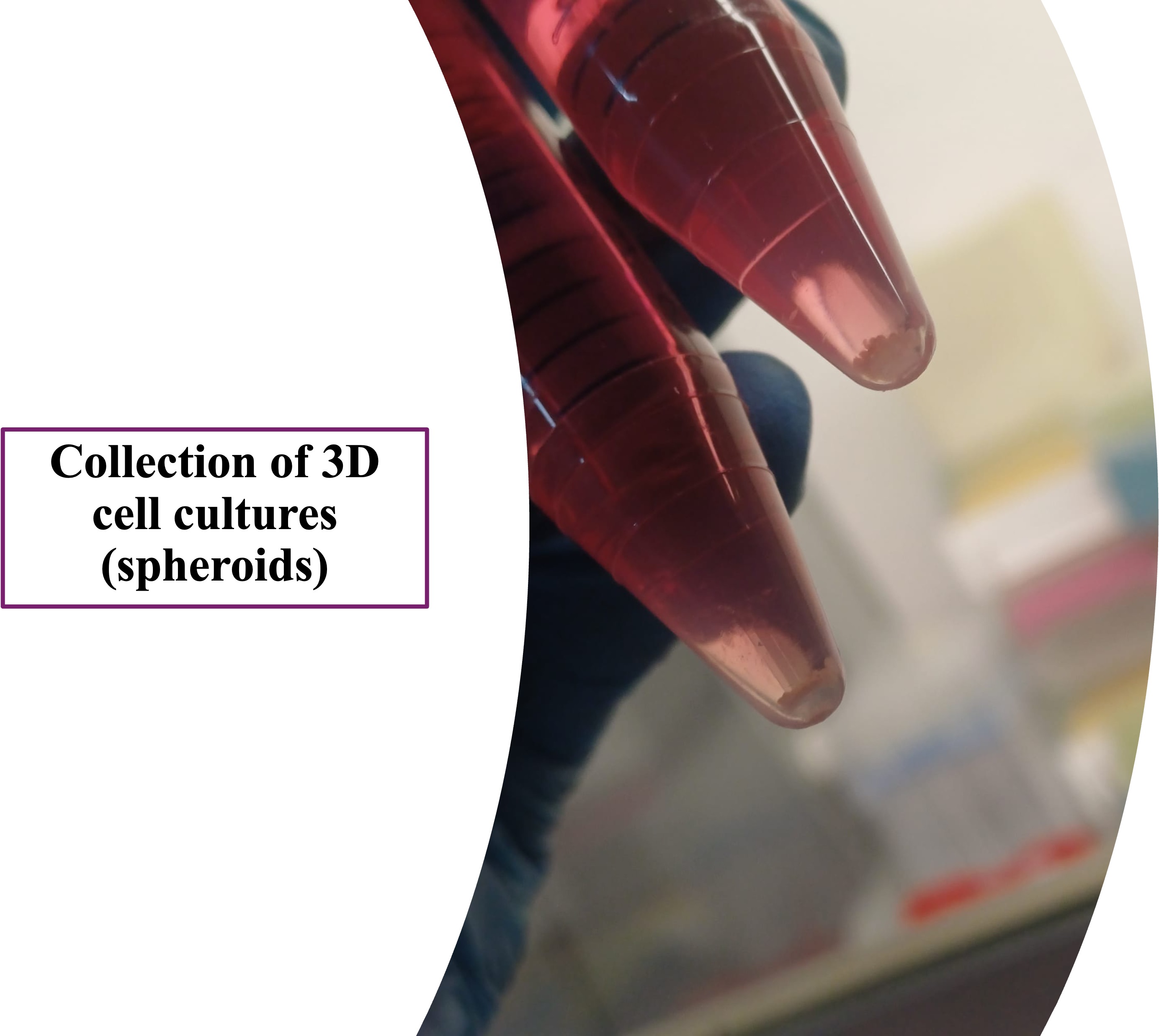

Supplement: Supplementary file 1 [file mps-09-00061-s001.zip › S5.jpg]

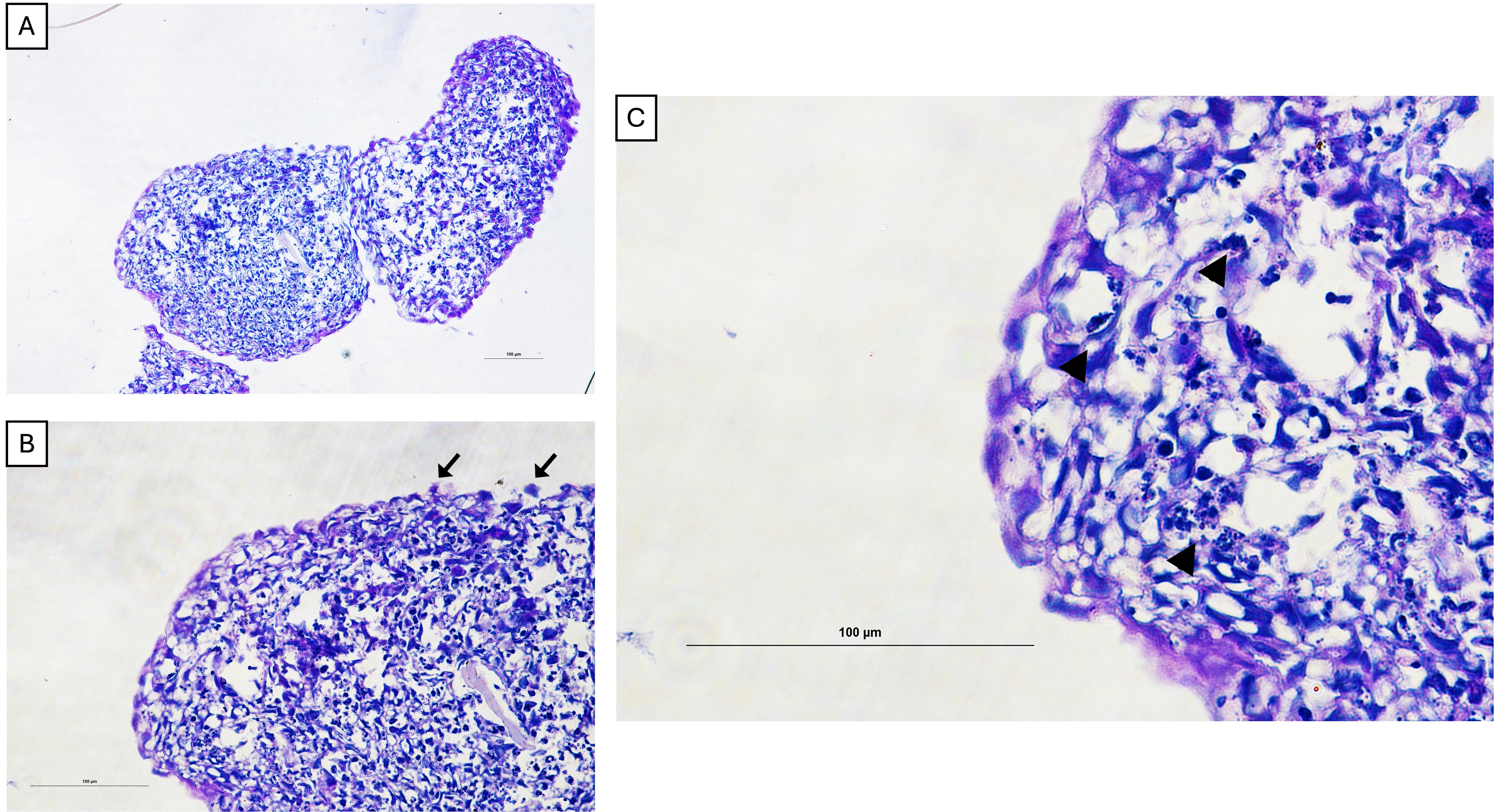

Supplement: Supplementary file 1 [file mps-09-00061-s001.zip › S6.jpg]
